# Spatial distribution and multilevel analysis of full vaccination coverage among children aged 12–23 months in Democratic Republic of Congo

**DOI:** 10.1016/j.gloepi.2026.100256

**Published:** 2026-03-08

**Authors:** Nigussie Adam Birhan, Abebew Aklog Asmare, Kefale Tilahun Getahun, Gedif Mulat Alemayehu, Zelalem Meraf Wolde, Denekew Bitew Belay

**Affiliations:** a Department of Statistics, College of Natural and Computational Science, Injibara University, Injibara, Ethiopia; b Department of Statistics, College of Natural and Computational Science, Mekdela Amba University, Tulu Awuliya, Ethiopia; c Department of Statistics, College of Science, Bahir Dar University, Bahir Dar, Ethiopia; d School of Health Systems and Public Health, Faculty of Health Sciences, University of Pretoria, Pretoria, South Africa

**Keywords:** Democratic Republic Congo, Full vaccination, Multilevel analysis, Spatial analysis

## Abstract

**Background:**

Vaccination is one of the most cost-effective public health interventions for reducing child morbidity and mortality. However, full vaccination coverage remains suboptimal in many low- and middle-income countries, including Democratic Republic Congo (DRC). This study aimed to assess spatial distribution and risk factors of full vaccination coverage among children aged 12–23 months in DRC.

**Methods:**

A cross sectional secondary data analysis on 2023/24 DRC Demographic and Health Survey data with a total weighted sample of 3897 children aged 12–23 months was used. Moran's I and Getis-Ord Gi* statistics was used to identify clustering patterns of full vaccination coverage. Multilevel analysis was used to examine factors associated with full vaccination coverage.

**Result:**

The prevalence of full vaccination was 14.9% (95% CI: 13.8, 16.0). Moran's I 0.4 (*p* value = 0.01) indicated spatial clustering of full vaccination coverage. Full vaccination coverage was associated with maternal age 25–34 years (AOR = 1.54, 95% CI: 1.06–2.24), secondary and above education (AOR = 1.84, 95% CI: 1.18–2.88), being married (AOR = 1.55, 95% CI: 1.15–2.09), rich household wealth (AOR = 1.64, 95% CI: 1.06–2.51), 1–7 antenatal care visits (AOR = 2.25, 95% CI: 1.40–3.62), health facility delivery (AOR = 3.34, 95% CI: 1.87–5.97), and rural residence (AOR = 0.59, 95% CI: 0.39–0.89).

**Conclusion:**

Full vaccination coverage in DRC is low and unevenly distributed; with cold spots in Mongala, part of Bas Uele and Tshuapa regions. Hence, targeted interventions focusing on identified cold spot areas, improving maternal education, expanding healthcare access, and promoting antenatal care and institutional delivery are essential to increase vaccine coverage.

## Introduction

Vaccination is considered to be one of the most successful public health strategies, having saved countless lives and reduced the morbidity and mortality of several diseases, thereby allowing the complete and healthy development of children, making it the right of every human being [1]. Childhood vaccination prevents an estimated 4–5 million deaths annually worldwide from diseases such as diphtheria, tetanus, pertussis, and measles and generating substantial economic returns [2].

Evidence drawn from 17 sub-Saharan African countries in 2023 shows that only a little over half of children aged 12–23 months (56.6%) were fully immunized, indicating that a substantial proportion of children in this age group remained not or partially vaccinated, despite the availability of routine immunization services [3].

Previous studies showed that 29% of deaths in children under the age of five were due to vaccine-preventable diseases (VPDs), and being fully immunized resulted in a 22% reduction in child mortality rates [4], [5].

According to the World Health Organization (WHO) and the United Nations Children's Fund (UNCF) reports, over 20 million children globally have not completed the full schedule of basic vaccinations. More than 60% of these unvaccinated or under-vaccinated children are concentrated in 10 low and middle income countries (LMICs). These countries are often disproportionately impacted by infectious diseases, a situation worsened by fragile healthcare systems or regional conflicts [6], [7].

Several factors hinder the sustainable delivery of vaccination services. These factors include conflict, inadequate investment in vaccination programs and shortages of vaccines and supplies. Conflict has been an ongoing determinant of lower vaccination coverage and an increased vaccine preventable disease outbreak [8], [9], [10], [11]. Providing childhood vaccination is challenging during and post conflict because of destruction of health care systems, shortage of vaccines and supplies, displacement and security issues [12]. Outbreaks of vaccine preventable diseases are frequently reported in conflict- affected countries [8], [13].

In this study, full vaccination is commonly defined as received the Bacille Calmette–Guérin (BCG), three doses of diphtheria-tetanus-pertussis (DTP) to prevent diphtheria, pertussis, and tetanus, three doses of polio vaccine (OPV), and one dose of the measles vaccine within the specified age [14]. Although there were significant differences across the various regions, the trend of vaccination coverage occasionally increased.

Previous researches have reported several factors associated with low vaccination coverage, including maternal education, access to healthcare, family socioeconomic status, location of childbirth, number of antenatal care visits, distance from health facilities, vaccination status, awareness of vaccination, child's sex, place of residence, religious affiliation, and exposure to mass media [15], [16], [17], [18]. Additionally, the low vaccination rates in LMICs are attributed to several factors, such as weak political commitment to vaccination programs, poor access to healthcare services, low public awareness, and a lack of proper education and understanding about vaccines among healthcare providers and caregivers, especially mothers [19]. However, the impact of these factors on achieving full vaccination has varied across different study settings. In DRC, where healthcare infrastructure remains fragile and many communities face barriers to healthcare access, understanding the local risk factors of poor vaccination coverage is essential for tailoring effective interventions.

To our knowledge, the risk (cold spot) areas for full vaccination coverage among children aged 12–23 months have not been identified in DRC. Identifying geographical variations in full vaccination coverage is very important to prioritize and design targeted prevention and intervention programs to improve vaccination coverage at the national level.

## Methods

### Study area

This study was focused on the Democratic Republic of Congo, the second-largest country in Africa after Algeria. The Democratic Republic of Congo stretches from the Atlantic Ocean to the eastern plateau, covering most of the Congo River basin. The country shares borders with nine neighboring countries, including the enclave of Cabinda. Located in Central Africa, the Democratic Republic of Congo is crossed by the equator and stretches across the Congo Basin [20].

### Data source and sampling method

The data utilized in this research were sourced from the 2023–2024 Democratic Republic of Congo Demographic and Health Survey dataset, marking the third DHS conducted in the Democratic Republic of Congo from October 2023 to January 2024. A total of 780 clusters were selected as primary sampling units, representing either neighborhoods in urban settings or villages in rural regions. Additionally, 26,452 households were chosen as secondary sampling units, with 7616 located in urban areas across 224 clusters and 18,836 in rural areas within 556 clusters. Out of the 26,347 households surveyed, 27,846 women aged 15–49 were eligible for the individual survey, and 27,583 of them participated, resulting in a response rate of 99.1% [21]. Following registration and the specification of the analysis purpose, the dataset was acquired from the Measure Demographic and Health Survey (DHS) website (http://www.dhsprogram.com). The kids' record dataset was used. A two-stage sampling design was implemented to provide balanced representation across urban and rural areas. In the first stage of sampling, enumeration areas were randomly selected, followed by the identification of households in the second stage. Children aged 12–23 months with complete information on vaccination status were included in the analysis. Records with missing outcome data were excluded prior to analysis. Hence, the final analysis was based on a weighted sample of 3897 children aged 12–23 months.

### Source population and study population

The source population consisted of all children in DRC who were between 12 and 23 months old at the time of the national survey. This study specifically included those children within this age range and whose data were collected during the data collection period.

### Variables of the study

#### Outcome variable

The outcome variable of the study was the complete basic childhood full vaccination status of children aged 12–23 months, categorized as a binary variable. A value of “yes” was assigned if a child had received one dose of Bacillus-Calmette-Guérin (BCG) vaccine at birth, three doses of Diphtheria-Pertussis-Tetanus (DPT) and polio vaccine and (at 6, 10, and 14 weeks, including oral polio vaccine at birth) vaccine, and one dose of Measles vaccine (at 9 months of age). A value of “no” was recorded if the child had not received all the recommended doses. Vaccination status was determined using written vaccination cards when available; if a card was not available, maternal recall was used, consistent with DHS standard procedures.

#### Independent variables

We included explanatory variables in the analysis, selected from the DHS dataset based on prior research and existing literature [22], [23], [24]. The independent variables were classified as individual level factors including child's sex, birth order, maternal age, maternal employment status, place of delivery, antenatal care visit (ANC), living children, birth order, household's wealth status, media exposure, size of child at birth, number of house hold member, number of children under five in the households, maternal education level, marital status, healthcare decision maker, distance to health facility, wanted pregnancy; community level variables include place of residence, community education level, community poverty and community media exposure level. Community media exposure was generated by aggregating individual-level access to radio, television, and newspapers within each cluster (enumeration area). Then, the variable was dichotomized into high with proportions above the median and those at or below the median as low category based on the national median value.

### Data management and analysis

The dataset was processed and examined using STATA 18 Software. Prior to conducting any statistical analysis, the data were adjusted with sampling weight (V005), to ensure the survey's representativeness and account for the sampling design, thereby achieving reliable statistical estimates. To investigate full vaccination coverage among children aged 12–23 months, descriptive statistics, multilevel logistic regression models and spatial analysis were employed. Descriptive statistics were utilized to outline the counts and proportions of children and their background characteristics related to full vaccination. A multilevel model is a statistical approach that accommodates independent variables at any hierarchical level and includes at least one random effect above the first-level group [25].

We applied a multilevel logistic regression model to assess the association between being full vaccination coverage among children aged 12–23 months and the predictors, and expressed these associations using odds ratios (ORs), which indicate the likelihood of the outcome occurring in one group compared to another.

Specifically, multilevel models were developed using a mixed effects modeling approach for the hierarchical structure of the data. In the DHS, children are nested within households, and households are nested within clusters (enumeration areas), meaning that children within the same cluster may share similar environmental, socioeconomic, and health-related exposures. Standard regression methods that ignore this clustering could underestimate standard errors and produce biased estimates. By using multilevel modeling, we were able to partition the variation into individual- and community-level components and obtain accurate estimates of both fixed effects of explanatory variables and random effects at the community level [26].

Bivariate analysis was conducted to assess associations between each independent variable and full vaccination. Those variables identified in this analysis were included in the multivariable analysis both in the individual and community level models. In the multivariable multilevel analysis, variable association with full vaccination coverage was interpreted using adjusted odds ratios (AOR) [27]. Before fitting the multivariable logistic regression model, multicollinearity among independent variables was assessed using the variance inflation factor and tolerance statistics. Variables with variance inflation factor > 10 were considered potentially collinear. No evidence of problematic multicollinearity was detected, so all candidate variables were included in the final model.

The models' comparison and fitness were evaluated using the Likelihood Ratio (LR) test and deviance (−2LR) values, as the models were nested. Consequently, the multilevel mixed-effects logistic regression analysis emerged as the best-fitting model due to its lowest deviance value.

### Model building

Four models were fitted. The first model (empty model) excludes independent variables in order to decompose the total variance into its cluster and country components which was used to check variation in community and provide evidence to assess random effects at the community level. Model I was the multivariable model adjustment for all individual-level independent variables and model II was adjusted for community-level factors. In model III, possible candidate variables from both individual and community-level variables were fitted with the full vaccination coverage among children aged 12–23 months**.**

### Parameter estimation method

The fixed effects (a measure of association) were used to estimate the association between the likelihood of full vaccination coverage and explanatory variables at both community and individual level and were expressed as odds ratio with 95% confidence interval. Regarding the measures of variation (random-effects), Community-level variance with standard deviation, intra cluster correlation coefficient (ICC), Proportional Change in Community Variance (PCV), and median odds ratio (MOR) was used.

The intra-class correlation indicates the proportion of the variance explained by the grouping structure in the population. When logistic model is used the residual at level one (child level) are assumed to follow the standard logistic distribution with mean 0 and variance $\frac{\pi^{2}}{3}=3.29.$ It is expressed as: $\mathrm{ICC}=\frac{\sigma_{\mu 0}^{2}}{\sigma_{\mu 0}^{2}+\frac{\pi^{2}}{3}}$ where, $\sigma_{\mu 0}^{2}\;$is the variance of the higher level (Community).

The aim of the median odds ratio is to translate the area level variance in the widely used odds ratio (OR) scale, which has a consistent and intuitive interpretation. The median odds ratio is defined as the median value the odds ratio between the area at the highest risk and the area at the lowest risk when randomly picking out two areas. The median odds ratio can be conceptualized as the increased risk that (in median) would have if moving to another area with a higher risk. It is computed by; $\mathrm{MOR}=\exp(\sqrt{\left(2\mathrm{xVA}\right)}\;x0.6745$).

Where; VA is the area level variance, and 0.6745 is the 75th centile of the cumulative distribution function of the normal distribution with mean 0 and variance 1 [28]. Whereas the proportional change in variance is calculated as.

PCV = [$\frac{\left(\mathrm{VA}-\mathrm{VB}\right)}{\mathrm{VA}}$]*100;

Where; VA = variance of the initial model, and VB = variance of the model with more terms.

### Spatial analysis

Spatial analysis was conducted using ArcGIS version 10.8. Spatial relationships between clusters were defined using a **queen contiguity spatial weight matrix**, in which clusters sharing either a common boundary or a vertex were considered neighbors. The spatial autocorrelation statistic (Global Moran's I) was used to evaluate the distribution pattern of full vaccination across the study area, specifically, whether it was clustered, dispersed, or randomly distributed. A Moran's I value close to −1 indicated a dispersed pattern, while a value near +1 suggested clustering. A value around 0 signified a random distribution [29].

### Hot spot analysis (Getis-Ord Gi* statistic)

To evaluate how spatial autocorrelation varied across the study area, the Getis-Ord Gi* statistic was used, calculating a Gi* value for each location. *Z*-scores were computed to identify areas with statistically significant hotspots and cold spots of full basic vaccination. High Gi* values indicated hotspot areas, while low Gi* values signified cold spots, based on the proportion of full basic vaccination among children aged 12 to 23 months [29].

## Results

### Socio demographic and socioeconomic characteristics of the study participants

This study included weighted sample of 3897 children aged 12–23 months; from this 2710(69.6%) resided in rural regions and 727(18.7%) women were illiterate. Out of all the participants in the study, 3378(86.7%) were born in a health facility, and 313(8.0%) were small size at birth [Table 1].

**Table 1:** Socio demographic characteristics of the study participants (*n* = 3897) in DRC for the year 2023/24.

Variable	Categories	Vaccination status		Total Frequency (%)
		Not fully vaccinated	Fully vaccinated	
		Frequency (%)	Frequency (%)	
age of mother	15–24	1140(34.4)	162(27.9)	1302(33.4)
	25–34	1425(43.0)	269(46.5)	1694(43.5)
	35–49	752(22.7)	148(25.6)	901(23.1)
mother education level	no education	690(20.8)	37(6.4)	727(18.7)
	primary	949(28.6)	117(20.2)	1066(27.4)
	secondary and above	1679(50.6)	425(73.4)	2104(54.0)
wealth index	poor	1607(48.5)	134(23.1)	1741(44.7)
	middle	664(20.0)	98(16.9)	762(19.6)
	Rich	1046(31.5)	347(60.0)	1394(35.8)
living children	1–2	1211(36.5)	247(42.6)	1457(37.4)
	3–4	998(30.1)	173(29.8)	1171(30.1)
	5 and above	1109(33.4)	160(27.7)	1269(32.6)
birth order	First	624(18.8)	140(24.1)	764(19.6)
	2–3	1035(31.2)	188(32.4)	1223(31.4)
	4–5	809(24.4)	149(25.7)	959(24.6)
	6 and above	849(25.59)	103(17.8)	952(24.4)
media exposure	no	2010(63.3)	227(39.2)	2327(59.7)
	Yes	1217(36.7)	353(60.9)	1570(40.3)
Number of house hold member	4 and less	732(22.1)	135(23.2)	866(22.2)
	5–8	1837(55.4)	304(52.4)	2140(54.9)
	above 8	749(22.6)	141(24.4)	890(22.9)
place of delivery	home	507(15.3)	12(2.1)	519(13.3)
	health facility	2810(84.7)	567(97.9)	3378(86.7)
Child birth size	small	244(7.3)	69(11.9)	313(8.0)
	average	1605(48.4)	263(45.3)	1868(47.9)
	large	1469(44.3)	248(42.8)	1716(44.1)
Under five children	One	937(28.23)	211(36.5)	1148(29.5)
	Two	1514(45.63)	239(41.3)	1753(45.0)
	Above two	867(26.13)	129(22.3)	996(25.6)
Healthcare decision maker	Respondent alone	285(10.1)	37(8.0)	322(9.8)
	Respondent and partner	1289(45.8)	251(54.0)	1540(47.0)
	Partner alone	1239(44.1)	176(38.0)	1416(43.2)
Wanted pregnancy	No	276(8.3)	62(10.7)	338(8.7)
	Yes	3041(91.7)	518(89.30)	3559(91.3)
mother employment status	Not employed	1177(35.5)	230(39.7)	1407(36.1)
	Employed	2140(64.5)	350(60.3)	2490(63.9)
ANC	No	534(17.3)	38(7.0)	572(15.8)
	1–7	2489(80.5)	490(91.7)	2979(82.2)
	8 and above	69(2.2)	7(1.3)	76(2.1)
Child age	12–18	2045(61.7)	380(65.5)	2425(62.2)
	19–23	1272(38.3)	200(34.5)	1472(37.8)
Child sex	Male	1685(50.8)	276(47.7)	1961(50.3)
	Female	1632(49.2)	304(52.3)	1936(49.7)
Distance to health facility	not big problem	2092(63.1)	445(76.8)	2536(65.1)
	big problem	1226(37.0)	134(23.3)	1360(34.9)
marital status	unmarried	1491(44.9)	231(39.9)	1722(44.2)
	married	1827(55.1)	348(60.1)	2175(55.8)
Type of place of residence	Urban	879(26.5)	308(53.1)	1187(30.5)
	Rural	2438(73.5)	272(46.9)	2710(69.6)
Community education level	Low	1742(52.5)	191(32.9)	1933(49.6)
	High	1575(47.5)	389(67.1)	1964(50.4)
Community poverty level	Low	1794(54.1)	370(63.8)	2164(55.5)
	High	1523(45.9)	210(36.2)	1733(44.5)
Community media exposure level	Low	1569(47.3)	141(24.4)	1710(43.9)
	High	1749(52.7)	438(75.6)	2187(56.1)

### Prevalence of full childhood vaccination coverage in DRC

The finding of this study revealed that prevalence of full vaccination coverage among children aged 12–23 months in Democratic Republic of Congo was 14.9% (95% CI: 13.8, 16.0). From the overall vaccine coverage, 35.6% was obtained from individuals' vaccine cards, while the remaining 64.4% was from maternal recall. The coverage of individual vaccines among children aged 12–23 months varied widely from BCG (69.2%) to Polio 3 (31.2%) (Table 2).

**Table 2:** prevalence of full vaccination coverage of children aged 12–23 months in DRC.

Type of vaccine	Frequency (%)	95% CI
BCG	2696(69.2)	67.7, 70.6
Polio at birth	2107(54.1)	52.5, 55.6
Polio 1	2668(68.5)	67.0, 69.9
Polio 2	2165(55.6)	54.0, 57.1
Polio 3	1217(31.2)	29.8, 32.7
DPT1	2592(66.5)	65.0, 68.0
DPT2	2287(58.7)	57.1, 60.2
DPT3	1803(46.3)	44.7, 47.8
Measles	2171(55.7)	54.2, 57.3
Overall	580(14.9)	13.8, 16.0

### Model comparisons in multilevel binary logistic regression analysis

The intra-class correlation coefficient (ICC) in the null model was 0.38, meaning that variations among DRC accounted for 38% of the variation in the full vaccination coverage. According to the final model's proportional change in variance (PCV), both individual- and community-level factors accounted for 46.74% of the variation in the full vaccination coverage. Significant variation in the full vaccination coverage across communities was confirmed by the model I median odds ratio, which was 3.90 and showed a significant clustering effect. In addition, we compared the four models using Deviance and Log-likelihood, and model III was the best-fitting model included in the analysis and utilized to interpret the results of this study. LR test also confirmed that Model III is significantly better than Model I (Table 3).

**Table 3:** Comprehensive comparison of models and outputs of fitness parameters for assessing full vaccination coverage of children aged 12–23 months in DRC.

Fitness parameter	Null model	Model I	Model II	Model III
Community level variance	2.04	1.11	1.45	1.09
ICC	0.38	0.25	0.30	0.25
MOR	3.90	2.73	3.14	2.70
PCV	Reference	45.81	29.62	46.74

Model fitness parameters				
Log- likelihood ratio (LLR)	−1398.35	−1025.65	−1349.70	−1021.24
Deviance	2796.70	2051.29	2699.4	2042.49
LRT(Likelihood ratio test)		χ^2^ = 216.88, *p*-value <0.001	χ^2^ = 119.58, p-value <0.001	χ^2^ = 133.08, p-value <0.001

### Factors associated with the full vaccination coverage

The results of the bivariate analysis showed significant explanatory variables that exhibited statistical significance in their unadjusted association with the full vaccination coverage (Table 1A). Hence, a multilevel multivariable logistic regression model was fitted by excluding household members, mother employment status, child age and child sex. Accordingly, maternal education, maternal age, wealth index, place of delivery, ANC, Distance to health facility, marital status, place of delivery and residence were significantly associated with full vaccination coverage in DRC (Table 4).

**Table 4:** Multilevel logistic regression analysis of determinants of full vaccination coverage of children aged 12–23 months in Democratic Republic Congo.

Variable	Categories	Model I AOR (95% CI)	Model II AOR (95% CI)	Model III AOR (95% CI
age of mother	15–24	1		1
	25–34	1.58(1.09, 2.29)		1.54 (1.06, 2.24)
	35–49	1.83(1.09, 3.07)		1.74(1.03, 2.99)
mother education level	no education	1		1
	primary	1.41(0.91, 2.18)		1.42(0.90, 2.23)
	secondary and above	1.93(1.26, 2.96)		1.84(1.18, 2.88)
wealth index	poor	1		1
	middle	1.29(0.91, 1.82)		1.26(0.87, 1.83)
	Rich	2.24(1.55, 3.25)		1.64(1.06, 2.51)
living children	1–2	1		1
	3–4	0.96(0.61, 1.52)		0.96(0.61, 1.51)
	5 and above	0.93(0.48, 1.80)		0.92 (0.48, 1.79)
birth order	First	1		1
	2–3	0.74(0.47, 1.19)		0.74 (0.47, 1.19)
	4–5	0.69(0.3, 1.31)		0.71(0.37, 1.34)
	6 and above	0.63(0.29, 1.39)		0.66 (0.30, 1.48)
media exposure	no	1		1
	Yes	1.20(0.90, 1.61)		1.10 (0.81, 1.51)
place of delivery	home	1		1
	health facility	3.49(1.96, 6.23)		3.34 (1.87, 5.97)
Child birth size	small	1		1
	Average	0.81(0.49, 1.31)		0.82(0.50, 1.33)
	Large	0.72(0.44, 1.17)		0.74 (0.46, 1.21)
Under five children	One	1		1
	Two	0.93(0.66, 1.30)		0.93 (0.67, 1.30)
	Above two	0.78(0.52, 1.18)		0.81(0.54,1.21)
Healthcare decision maker	Parent alone	1		1
	Respondent and partner	1.27(0.96, 1.67)		1.27(0.96, 1.67)
	Respondent alone	1.12(0.72, 1.73)		1.10(0.71, 1.70)
Wanted pregnancy	No	1		1
	Yes	1.06(0.61, 1.84)		1.05(0.60, 1.83)
ANC	No	1		1
	1–7	2.21(1.38, 3.55)		2.25 (1.40, 3.62)
	8 and above	2.08(0.81, 5.35)		2.18 (0.85, 5.60)
Distance to health facility	not big problem	1		1
	big problem	0.70(0.53, 0.93)		0.72(0.54, 0.95)
marital status	unmarried	1		1
	married	1.55(1.15, 2.09)		1.55 (1.15, 2.09)
Type of place of residence	Urban		1	1
	Rural		0.29(0.21, 0.42)	0.59(0.39, 0.89)
Community education level	Low		1	1
	high		1.16(0.83,1.62)	0.95 (0.66, 1.36)
Community poverty level	Low		1	1
	High		0.94(0.70, 1.27)	0.89(0.63, 1.24)
Community media exposure level	Low		1	1
	High		1.61(1.15, 2.25)	1.17(0.80, 1.71)

Children born to mothers aged 25–34 years and above 34 years had a 54% higher odds (AOR = 1.54, 95%CI: 1.06,2.24) and a 74% higher odds (AOR = 1.74; 95%CI: 1.03, 2.92) of full vaccination coverage as compared to children born to mothers aged less than 25 years respectively. Children from rich households had 64% higher odds of having the full vaccination coverage than children from poor households (AOR = 1.64; 95%CI: 1.06, 2.51). Institutional delivery was another significant factor; children born in health facilities had higher odds of full vaccination (AOR = 3.34; 95%CI: 1.87, 5.97). Children whose mothers utilized 1–7 antenatal care were found to have more than two times the odds of being fully vaccinated (AOR = 2.25, 95%CI: 1.40, 3.62) compared to those whose mothers did not attend antenatal care. Mothers with secondary and above educations had 84% (AOR = 1.84; 95%CI: 1.18, 2.88) higher odds of fully vaccinated their children than the uneducated mothers. The odds of full vaccination coverage among children 12–23 months of age from mothers/caregivers who reside in the rural was 41% lower than that of deviant mothers/caregivers who reside in the urban (AOR = 0.59; 95%CI: 0.39, 0.89) (Table 4).

### Global spatial autocorrelation analysis of full childhood vaccination coverage among children aged 12 to 23 months

The spatial analysis using Moran's I revealed a Moran's Index of 0.4, a z-score of 2.5, and a p-value of 0.01, indicating a highly clustering of complete basic childhood vaccination coverage across regions in DRC.

### Getis-Ord Gi* of full childhood vaccination coverage among children aged 12 to 23 months

**Fig. 1:**
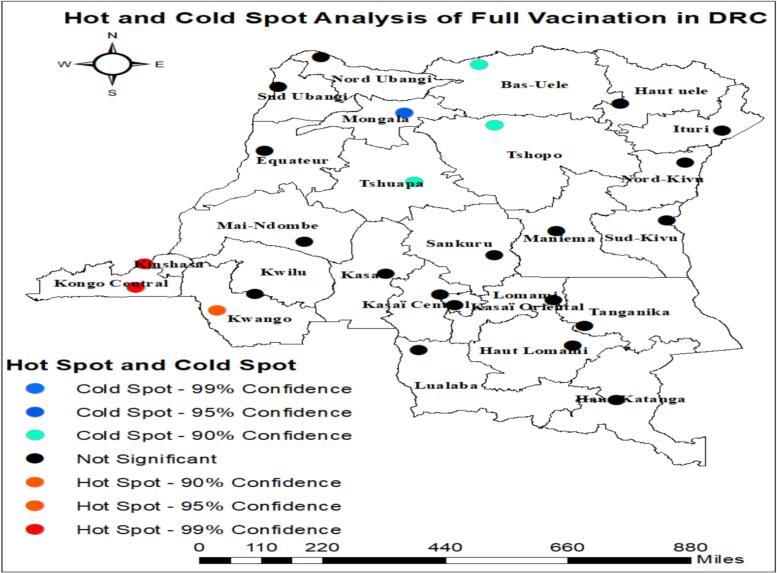
Hot spot analysis of full childhood vaccination coverage among children aged 12 to 23 months.

The hotspot analysis of full vaccination revealed significant clusters across DRC. The prominent cold spots were concentrated in the northern and northeastern provinces. Mongala and Nord Ubangi were classified as strong cold spots at the 99% confidence level, while Bas-Uele, Tshuapa, Tshopo, and Ituri showed statistically significant low-value clustering at the 90–95% confidence levels. These cold spot areas consistently exhibit lower vaccination rates. However, high-value clusters of full vaccination were detected in the southwestern part of the country. Kongo Central and portions of Kinshasa emerged as strong hot spots at the 99% confidence level, indicating markedly higher vaccination performance relative to neighboring provinces. A moderate hot spot was identified in Kwango (95% confidence). The presence of these hot spots likely reflects improved service availability, stronger health infrastructure, and increased urbanization that facilitate vaccine uptake. Large parts of the country indicating no clear clustering (Fig. 1).

### Spatial interpolation of full vaccination in Democratic Republic Congo

**Fig. 2:**
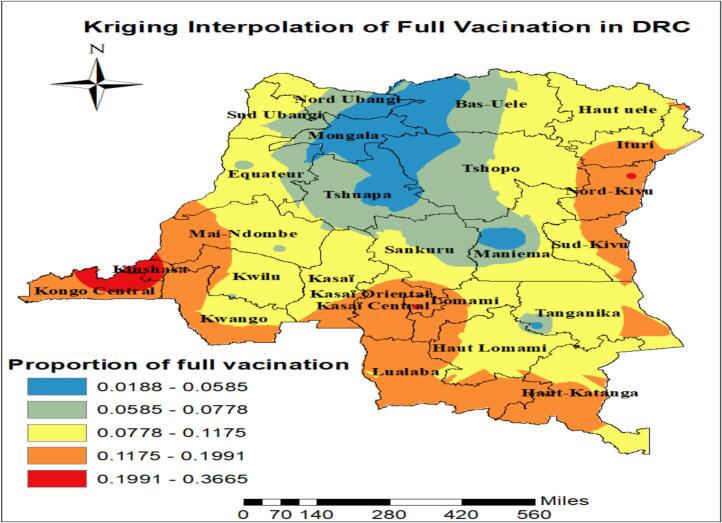
Kriging interpolation of full vaccination in Democratic Republic Congo based on CD 2023–24 DHS.

Kriging spatial interpolation illustrates the spatial distribution of full vaccination across Democratic Republic Congo. The lowest proportions (1.88%–5.85%) are concentrated in central region such as Mongala, part of Bas Uele and Tshuapa. The highest proportions (19.91%–36.65%) are observed in western regions such as Kongo Central and Kinsha. Overall, full vaccination prevalence decrease from West to North areas, indicating the need for targeted maternal and child health interventions in low-risk region (Fig. 2).

## Discussion

The prevalence of full vaccination coverage in DRC was found to be 14.9% (95% CI: 13.8, 16.0) based on the CD 2023–24 DHS. This result is lower than the coverage reported in Nigeria (23.3%) based on 2018 DHS [24], in the DRC-DHS 2007 (30.7%), DRC-MICS 2010 (48.6%), DRC-DHS 2013 45.3%), and DRC-MICS 2017 (21.8%) among one-year-olds [30]. Moreover, it is below the 90% coverage recommended by the WHO [1]. These low rates may be attributed to less effective health systems, limited community outreach, weak political commitment, and poor vaccination integration with other health services. The spatial distribution of full vaccination coverage was non-random across the country, with global spatial autocorrelation statistics (Moran's *I* = 0.4, *p*-value = 0.01), indicating significant spatial clustering of full vaccination coverage among children aged 12–23 months in the DRC. Full immunization coverage was highest among children from the provinces of Kongo-Central, Kinshasa and Kwango. However, the coverage is low in provinces such as Mongala, Nord Ubangi, Bas-Uele, Tshuapa, Tshopo, and Ituri have inadequate health infrastructure, local conflicts, or variations in community awareness, and should be prioritized in public health strategies [31]. The vaccination status was determined using maternal recall was 64.4% which reliance on maternal recall may introduce misclassification, particularly for multi-dose vaccines, potentially affecting coverage estimates. Furthermore, differences in ascertainment methods across surveys may contribute to the observed decline in reported coverage over time and transparent reporting of the source of vaccination information is essential for accurate interpretation. Therefore, public health officials should take all necessary measures to halt the low clustering of full vaccination among children aged from 12 to 23 months.

Maternal education was strongly associated with full vaccination coverage. Children whose mothers had higher levels of education had more likely of being fully vaccinated compared to those whose mothers had no education. This aligns with existing literature showing that educated mothers are better equipped to understand health information, access services, and follow vaccination schedules [32], [33].

Children from rich households had higher odds of being fully vaccinated than those from the poor household. This finding is in line with previous study [34], [35]. This might be explained by better access to healthcare facilities, transportation, and health information which tend to improve with economic standing [34].

Antenatal care visits during pregnancy was predictor of childhood full vaccination coverage. Children whose mothers attended ANC were more likely to be in full vaccination compared to those whose mothers did not utilize these care visits. This finding aligns with previous research [36], [37], which highlights that antenatal care play crucial roles in ensuring higher rates of childhood vaccination. Health institutions contribute to improved vaccination outcomes by providing immediate vaccination services, enhancing parental education [38], ensuring structured follow-up care, integrating vaccinations into comprehensive care plans [39] and enabling early detection and management of health issues.

The study also found that children born at health institutions are more likely to be in full vaccination compared to those born at home. This study supported by existing research [40], [41]. This might be health facilities provide immediate vaccination services, offer educational programs that increase parental awareness, ensure structured follow-up care, integrate vaccinations into antenatal and postnatal care, reduce barriers to accessing healthcare, and enable early detection of health issues [42].

Children of older women (25–34 years) and (35–49 years) are more likely to be fully immunized as compared to children of young mothers (15–24 years). This is in line with other studies [43], [44]. This might be **explained by the fact that older mothers tend to have greater maternal experience, better knowledge of child health services, and increased autonomy in healthcare decision-making** which can positively influence childhood immunization uptake [45].

### Strengths and limitations of the study

This study has several strengths that enhance its validity and relevance. It utilized nationally representative data from the recent DRC Demographic and Health Survey, ensuring generalizability to the broader population. The application of advanced spatial analysis techniques and multilevel logistic regression provided a robust understanding of both geographic distribution and multilevel determinants of full vaccination among children. Additionally, the large sample size strengthened the statistical power, and the policy-relevant findings offer practical implications for targeted public health interventions. However, the study is not without limitations. Its cross-sectional design limits causal inference. Second, vaccination status was partly based on maternal recall when vaccination cards were unavailable, which introduces potential recall. Third, although multilevel modeling was applied, residual confounding may persist due to unmeasured contextual factors such as local conflict intensity, security conditions, health system capacity, and service accessibility, which are not captured in DHS data.

## Conclusion

The full vaccination coverage in DRC is low and unevenly distributed, with cold spots in Mongala, part of Bas Uele and Tshuapa regions. Maternal age, maternal education, place of delivery, ANC visit, marital status, distance to health facility, and residence were associated with full childhood vaccination coverage among children aged 12–23 months in Democratic Republic of Congo. Hence, targeted interventions focusing on identified cold spot areas, improving maternal education, expanding healthcare access, promoting antenatal care and institutional delivery are essential to increase vaccine coverage and equity. Moreover, provincial can use spatial mapping results to guide micro-planning, resource allocation, and supervision activities.

## Declaration of generative AI and AI-assisted technologies in the manuscript preparation process

During the preparation of this work the author(s) used *ChatGPT (OpenAI)* in order to language editing. After using this tool/service, the author(s) reviewed and edited the content as needed and take(s) full responsibility for the content of the published article.

## Clinical trial number

Not applicable.

## Authors contribution

NAB involved in the study design, performed the data extraction, and analyzed and drafted the manuscript; GMA performed the data extraction; KTG and ZMW involved in analyzed and reviewed the manuscript; AAA and DBB involved in critically revising and reviewing the whole manuscript. All authors have read critically and approved the final manuscript.

## CRediT authorship contribution statement

**Nigussie Adam Birhan:** Writing – review & editing, Writing – original draft, Conceptualization. **Abebew Aklog Asmare:** Methodology, Formal analysis, Conceptualization. **Kefale Tilahun Getahun:** Writing – original draft, Software. **Gedif Mulat Alemayehu:** Writing – review & editing, Data curation. **Zelalem Meraf Wolde:** Writing – original draft, Visualization. **Denekew Bitew Belay:** Writing – review & editing.

## Consent for publication

Not applicable.

## Ethics approval and consent to participate

This research is based on publicly available, anonymous secondary data from the Demographic and Health Surveys (DHS) Program. The DHS data collection techniques were evaluated and approved by the ICF Institutional Review Board (IRB) in the United States and the Department of Health and Human Services' rules for human subject protection (45 CFR 46). All DHS surveys required informed consent, and confidentiality was rigorously protected. Participants aged ≤16 years provided parental or guardian consent. We obtained permission to use the data from the DHS Program https://dhsprogram.com/ and this secondary analysis did not require any additional ethical approval. We confirm that all methods were performed in accordance with the relevant guidelines and regulations and that this study adheres to the ethical principles of the Declaration of Helsinki.

## Funding

The authors received no specific funding for this work.

## Funding

This research received no specific grant from any funding agency in the public, commercial, or not-for-profit sectors.

## Declaration of competing interest

The authors declare no competing interests.

## Data Availability

The data used in this study is from demographic and health survey data which can be available via reasonable request from their website at DHS repository, https://www.dhsprogram.com.

## Appendix A Supplementary data

Supplementary material Image 1
