# Supplementary material for: Spatial distribution and multilevel analysis of full vaccination coverage among children aged 12–23 months in Democratic Republic of Congo
Source: Glob Epidemiol. 2026 Mar 8;11:100256. doi: 10.1016/j.gloepi.2026.100256 (PMC12995467; doi:10.1016/j.gloepi.2026.100256)
Supplement: Supplementary file 1 — Supplementary material [file mmc1.docx]

Table 1A. unweight frequency characteristics of the study participants in DRC for the year 2023/24

| **Variable** | **Categories** | **Vaccination status** | | **Total Frequency (%)** | **COR(95%CI)** | **P value** |
| --- | --- | --- | --- | --- | --- | --- |
|  |  | **Not fully Frequency (%)** | **Fully**  **Frequency (%)** |  |  |  |
| **Age of mother** | 15–24 | 1330(35.7) | 149(31.2) | 1479(35.2) | 1 | – |
|  | 25–34 | 1514(40.6) | 224(46.9) | 1738(41.3) | 1.32 (1.06–1.65) | 0.01 |
|  | 35–49 | 886(23.8) | 105(22.0) | 991(23.6) | 1.06 (0.81–1.38) | 0.7 |
| **Mother education level** | No education | 832(22.3) | 45(9.4) | 877(20.8) | 1 | – |
|  | Primary | 1216(32.6) | 118(24.7) | 1334(31.7) | 1.79 (1.26–2.56) | 0.001 |
|  | Secondary+ | 1682(45.1) | 315(65.9) | 1997(47.5) | 3.46 (2.51–4.78) | 0.0001 |
| **Wealth index** | Poor | 2154(57.8) | 160(33.5) | 2314(55.0) | 1 | – |
|  | Middle | 816(21.9) | 96(20.1) | 912(21.7) | 1.58 (1.21–2.07) | 0.001 |
|  | Rich | 760(20.4) | 222(46.4) | 982(23.3) | 3.93 (3.16–4.90) | 0.0001 |
| **Living children** | 1–2 | 1370(36.7) | 191(40.0) | 1561(37.1) | 1 | – |
|  | 3–4 | 1105(29.6) | 151(31.6) | 1256(29.9) | 0.98 (0.78–1.23) | 0.9 |
|  | ≥5 | 1255(33.7) | 136(28.5) | 1391(33.1) | 0.78 (0.62–0.98) | 0.03 |
| **Birth order** | First | 675(18.1) | 111(23.2) | 786(18.7) | 1 | – |
|  | 2–3 | 1166(31.3) | 152(31.8) | 1318(31.3) | 0.79 (0.61–1.03) | 0.08 |
|  | 4–5 | 894(24.0) | 113(23.6) | 1007(23.9) | 0.77 (0.58–1.02) | 0.07 |
|  | ≥6 | 995(26.7) | 102(21.3) | 1097(26.1) | 0.62 (0.47–0.83) | 0.001 |
| **Media exposure** | No | 2552(68.4) | 229(47.9) | 2781(66.1) | 1 | – |
|  | Yes | 1178(31.6) | 249(52.1) | 1427(33.9) | 2.36 (1.94–2.85) | 0.0001 |
| **Household members** | <5 | 849(22.8) | 110(23.0) | 959(22.8) | 1 | – |
|  | 5–8 | 2048(54.9) | 256(53.6) | 2304(54.8) | 0.96 (0.76–1.22) | 0.8 |
|  | >8 | 833(22.3) | 112(23.4) | 945(22.5) | 1.04 (0.81–1.37) | 0.7 |
| **Place of delivery** | Home | 829(22.2) | 18(3.8) | 847(20.1) | 1 | – |
|  | Health facility | 2901(77.8) | 460(96.2) | 3361(79.9) | 7.30 (4.53–11.77) | 0.0001 |
| **Child birth size** | Small | 276(7.4) | 53(11.1) | 329(7.8) | 1 | – |
|  | Average | 1690(45.3) | 209(43.7) | 1899(45.1) | 0.64 (0.46–0.89) | 0.008 |
|  | Large | 1764(47.3) | 216(45.2) | 1980(47.1) | 0.64 (0.46–0.88) | 0.007 |
| **Under-five children** | One | 1024(27.5) | 159(33.3) | 1183(28.1) | 1 | – |
|  | Two | 1713(45.9) | 203(42.5) | 1916(45.5) | 0.76 (0.61–0.95) | 0.02 |
|  | Three and above | 993(26.6) | 116(24.3) | 1109(26.4) | 0.75 (0.58–0.97) | 0.03 |
| **Healthcare decision maker** | Respondent alone | 336(9.0) | 39(8.2) | 375(8.9) | 1 | – |
|  | Respondent and partner | 1268(34.0) | 194(40.6) | 1462(34.7) | 1.32 (0.92–1.90) | 0.1 |
|  | Partner alone | 2126(57.0) | 245(51.2) | 2371(56.4) | 0.99 (0.69–1.42) | 1 |
| **Wanted pregnancy** | No | 324(8.7) | 29(6.1) | 353(8.4) | 1 | – |
|  | Yes | 3406(91.3) | 449(93.9) | 3855(91.6) | 1.47 (0.99–2.18) | 0.05 |
| **Mother employment status** | Not employed | 1328(35.6) | 166(34.7) | 1494(35.5) | 1 | – |
|  | Employed | 2402(64.4) | 312(65.3) | 2714(64.5) | **1.04 (0.85–1.37)** | **0.7** |
| **ANC visit** | No | 752(20.2) | 31(6.5) | 783(18.6) | **1** | **–** |
|  | 1-7 | 2912(78.1) | 435(91.0) | 3347(79.5) | 3.62 (2.50–5.26) | 0.0001 |
|  | 8 and above | 66(1.8) | 12(2.5) | 78(1.9) | 4.41 (2.16–8.99) | 0.0001 |
| **Child age** | 12–18 | 2317(62.1) | 300(62.8) | 2617(62.2) | 1 | – |
|  | 19–23 | 1413(37.9) | 178(37.2) | 1591(37.8) | 0.97 (0.80–1.18) | 0.8 |
| **Child sex** | Male | 1868(50.1) | 246(51.5) | 2114(50.2) | 1 | – |
|  | Female | 1862(49.9) | 232(48.5) | 2094(49.8) | 0.95 (0.78–1.14) | 0.6 |
| **Distance to HF** | Not big problem | 2098(56.3) | 341(71.3) | 2439(58.0) | 1 | – |
|  | Big problem | 1632(43.8) | 137(28.7) | 1769(42.0) | 0.52 (0.42–0.64) | 0.0001 |
| **Residence** | Urban | 841(22.6) | 238(49.8) | 1079(25.6) | 1 | – |
|  | Rural | 2889(77.5) | 240(50.2) | 3129(74.4) | 0.29 (0.24–0.36) | 0.0001 |
| **Marital status** | Unmarried | 1595(42.8) | 170(35.4) | 1765(41.9) | 1 | – |
|  | Married | 2135(57.2) | 308(64.4) | 2443(58.1) | 1.35 (1.11–1.65) | 0.003 |
| **Community media exposure** | Low | 2003(53.7) | 155(32.4) | 2158(51.3) | 1 | – |
|  | High | 1727(46.3) | 323(67.6) | 2050(48.7) | 2.42 (1.97–2.96) | 0.0001 |
| **Community education** | Low | 1995(53.5) | 178(37.2) | 2173(51.6) | 1 | – |
|  | High | 1735(46.5) | 300(62.8) | 2035(48.4) | 1.94 (1.59–2.36) | 0.0001 |
| Community poverty | Low | 1995(53.5) | 178(37.2) | 2173(51.6) | 1 | – |
|  | High | 1735(46.5) | 300(62.8) | 2035(48.4) | 0.77 (0.63–0.93) | 0.006 |
